# SUPPORTING PERSONS LIVING ALONE WITH DEMENTIA: FINDINGS FROM A PILOT STUDY

**DOI:** 10.1093/geroni/igad104.1898

**Published:** 2023-12-21

**Authors:** Allison Gibson, Natalie Pope, Mohammad Hossain

**Affiliations:** Saint Louis University, St. Louis, Missouri, United States; University of Kentucky, Lexington, Kentucky, United States; University of Kentucky, Lexington, Kentucky, United States

## Abstract

Older adults living alone with suspected cognitive impairment often have little-to-no support for conducting daily activities and may be more isolated from formal sources of support and healthcare services. As a result, many individuals living alone may receive delayed diagnosis of dementia compared to those that reside with others. The purpose of this study was to explore an innovative pilot program of case management services, Services to Age in Your Home (STAY Home), with local paramedicine services (interdisciplinary team of social work, law enforcement, and paramedics) that links this population to healthcare and social services through case management services in an urban area. While many participants were willing to engage in the pilot program, access to healthcare and social services remained challenging due to social service program waitlists, discomfort using formal services, distrust of losing autonomy, and limited financial resources contributed to barriers in pilot implementation. A biopsychosocial survey identified individuals needing cognitive testing (88.9%), support in managing medications and medication adherence (88.9%), unmet financial needs (77.8%), unmet healthcare needs (77.8%), difficulty managing finances (66.7%), challenges accessing transportation (55.6%), and unmet assistance needs for their ADLs (44.4%). Most individuals also reported limited social supports (i.e., neighbors, out-of-town relatives) with most relying on local formal services to address emergency needs. This presentation will include a discussion of ethical challenges for both conducting research as well as providing services to this low-resource population.

